# Gas permeation and microstructure of reduced graphene oxide/polyethyleneimine multilayer films created *via* recast and layer-by-layer deposition processes†

**DOI:** 10.1039/d1ra09205g

**Published:** 2022-02-25

**Authors:** Chongshan Yin, Xuan Du, Zhi Ding, Qing Zeng, Xi Li, Chunqing He, Bangyun Xiong, Jingjing Li, Yawei Zhou

**Affiliations:** Hunan Provincial Key Laboratory of Flexible Electronic Materials Genome Engineering, School of Physics and Electronic Science, Changsha University of Science and Technology Changsha 410114 China c.sh.yin@foxmail.com; Key Laboratory of Nuclear Solid State Physics Hubei Province, School of Physics and Technology, Wuhan University Wuhan 430072 China; School of Materials Science and Energy Engineering, Foshan University Foshan 528000 China; State Key Laboratory of Intense Pulsed Radiation Simulation and Effect, Northwest Institute of Nuclear Technology Xi'an 710024 China

## Abstract

Nowadays, graphene/polymer composite films with multilayer structure have attracted significant attention for gas barrier application. In this study, a series of reduced graphene oxide/polyethyleneimine (RGO/PEI) composite films were created *via* recast and layer-by-layer deposition processes. By using the recast process, the myriad PEI molecules in the precursor solution (the PEI : GO feeding ratio is 0.02 : 0.1, 0.05 : 0.1, 0.1 : 0.1, 0.3 : 0.1 and 0.5 : 0.1) ensure more effective reduction and surface modification of the graphene oxide (GO) sheets, while the undesirable free PEI molecules are eventually removed *via* a filtration process. Then, the RGO/PEI composite films were synthesized on PET substrate using a layer-by-layer assembly. The resulting films show a homogeneous and compact brick-wall structure with excellent gas barrier properties. Barriers against water vapor, nitrogen/oxygen, and carbon dioxide require different content of PEI in the composite film for optimal performance; the ideal values are 19.7, 23.8, and 24.1 wt%, respectively. These values are much lower compared with previously reported studies. Further, the permeability, free volumes, component ratio, morphology, and density of the RGO/PEI composite films have been carefully investigated and discussed. The results revealed that the mechanism behind the excellent gas barrier property of the RGO/PEI composite films is a synergistic effect created by the combination of the brick-wall structure, the small free volume holes, the suitable PEI content (ranging from 19.7 wt% to 24.1 wt%), the high density, and the hydrophobicity.

## Abstract 2

Gas barrier property and microstructure of reduced graphene oxide/polyethyleneimine multilayer films created *via* recast and layer-by-layer deposition processes.

## Graphical abstract

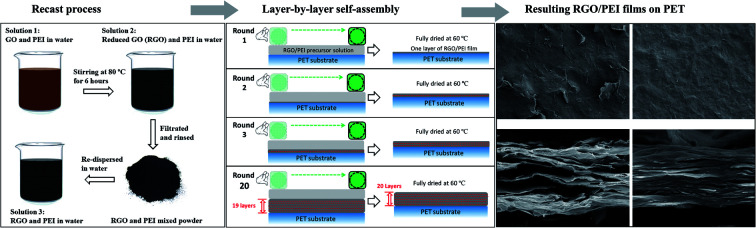

## 1 Introduction

Both reactive gases such as oxygen as well as water molecules in the natural environment cause the degradation of food, damage microelectronic devices, and reduce the lifetime and stability of metal components.^1^ Over years, packaging films designed to protect these items from reactive gases and water molecules have attracted strong scientific and technological interest.^2–4^ Many barrier solutions have been successfully developed in the past 20–30 years, including metallized plastic films,^5^ SiO_*x*_,^6,7^ and composite films.^2–11^ Most inorganic films show excellent intrinsic barrier behavior, but many of them tend to be compromised by pinholes or defects after being bent or stretched.^5–8^ Polymer-based barrier films are more flexible and have better mechanical properties, but their microstructure and barrier property are sensitive to the external environment, resulting in degradation over time.^9–11^ More recently, two-dimensional inorganic plates/polymer composite films have attracted much attention. During layer-by-layer assembly the electrostatic interaction among the components forces the platelets to deposit in a highly oriented fashion, forming a multilayer brick-wall structure.^12,13^ Within these films, gas molecules must avoid the brick part and therefore take a more tortuous path to permeate the film, greatly reducing gas permeability.^14^ A series of two-dimensional inorganic plates/polymer composite films with remarkable gas barrier properties have been reported.^15–27^ Among the types of two-dimensional inorganic plates that are available, graphene, or reduced graphene oxide (RGO), is one of the most preferred options, because it has high surface-to-volume ratio and excellent mechanical properties, as well as being hydrophobic and impermeable to most gases.^15–35^ For example, RGO/polyethyleneimine composite films with a brick-wall multilayer structure show excellent barrier behavior, high stability, and enhanced mechanical properties, making them suitable for barrier applications.^17–19^

However, the presence of hydrophilic polymers causes plasticization in the presence of moisture, resulting in rearrangement of the microstructure and increase in the gas permeability of the composite films.^36,37^ In order to reduce this moisture sensitivity, hydrophobic inorganic plates (such as graphene and RGO) and less hydrophilic polymers are utilized.^36–39^ To optimize the performance of composites, specific component ratios and processing conditions are required, and intensive studies could be very helpful in determining the optimal parameters.^15–34^ For a composite polymer film, the microstructure and interactions among components can be extremely complex,^40–43^ as well as the dynamics of gas permeation within it.^44–46^ Previous studies have mainly focused on the performance of these films.^47–51^ Further study into the important question of how the performance of barrier films is influenced by their microstructure and free volumes is needed.

In this work, a series of RGO/PEI composite films have been prepared *via* recast and layer-by-layer deposition processes. The influence of the preparation method on the structure, component ratio, and gas permeability of RGO/PEI composite films has been studied, and a related mechanism has been discussed. Further, a positron annihilation lifetime spectroscopy (PALS) method has been employed to analyze the free volumes within the samples.

## 2 Experimental

### 2.1 Materials

Single-side-polished (100) silicon wafers were used as substrates for the XRD measurement. Silicon wafers were cleaned with a 3 : 1 ratio of 30% hydrogen peroxide/99% sulfuric acid, and rinsed with acetone, and then stored in deionized water. A commercial poly(ethylene terephthalate) (PET) film (35 μm, Toray, Japan) was used as substrates for gas permeability and SEM measurements. PET substrate was rinsed with deionized water and methanol, corona-treated, and then a small amount of PEI solution (0.5 mL, 0.1 mg mL^−1^) was coated on the PET substrate. The corona treatment lasts a minute, and the frequency of the current is 20 kHz and the tension applied to the electrodes is 16 kV. Both the corona treatment and the thin PEI coating (∼4.9 × 10^−3^ μm) were expected to improve adhesion ability of the substrates. A commercial polystyrene (PS) film was used as substrate for cross-section SEM measurement. PS substrate was rinsed with deionized water and methanol and then corona-treated. The graphene oxide sheets (maximum outer diameter: 0.5–3 μm, number of sheets: <3) and graphene sheets (maximum outer diameter: 0.5–3 μm, number of sheets: <10) were purchased from Chengdu Organic Chemicals Co. Ltd (Chinese Academy of Sciences). A TEM morphology for the GO sheets was shown in Fig. S1.† Branched polyethyleneimine (PEI, MW = 10 000 g mol^−1^, 99 wt%) was purchased from Aladdin-e.com, China. Deionized water (purified with Milipore, resistivity = 18 MΩ cm^−1^) was used in this study. All other solvents and chemicals were reagent grade and were used as received.

### 2.2 Preparation of the reduced graphene oxide/polyethyleneimine (RGO/PEI) mixture

**Fig. 1:**
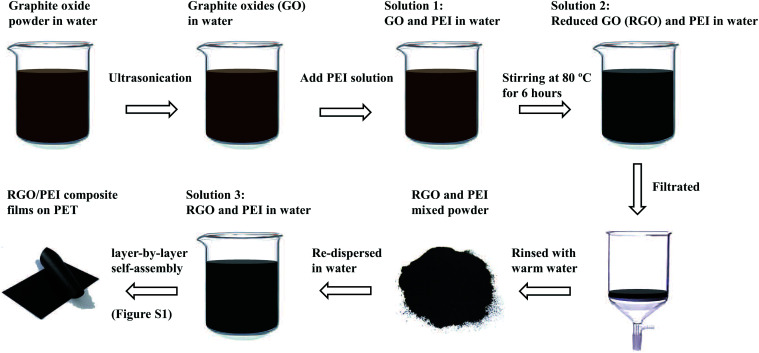
Schematic procedure to prepare the RGO/PEI composite films.

Fig. 1 depicts a schematic of the procedure to prepare the RGO/PEI solution and RGO/PEI composite films. Firstly, graphite oxide powder was dispersed in deionized water with a concentration of 0.1 mg mL^−1^, then subjected to ultrasonication (10 W) for 10 min to obtain the single-layer graphite oxides (GO). The resulting GO dispersion was mixed with a certain amount of PEI solution to obtain the solution 1 (the PEI: GO feeding ratio is 0.02 : 0.1, 0.05 : 0.1, 0.1 : 0.1, 0.3 : 0.1 and 0.5 : 0.1, respectively). The solution 1 was sealed and continuously stirred at 80 °C for 6 hours, to obtain the solution 2. This treatment is according a previously reported procedure,^17^ and is used to transform the GO into RGO and to bond some PEI molecules on the RGO sheets. The resulting solution 2 was filtrated with a cellulose acetate membrane (0.2 μm for pore size), and rinsed with warm deionized water (40 °C) for several times. The superfluous free PEI molecules are presumed to be removed along with the filter liquor, leaving the majority of the covalently bonded PEI molecules and a portion of physisorbed PEI molecules within the RGO/PEI composite. This process ensures that the GO sheets have sufficient contact with the myriad PEI molecules (the PEI: GO feeding ratio is 0.02 : 0.1, 0.05 : 0.1, 0.1 : 0.1, 0.3 : 0.1 and 0.5 : 0.1) in solution 2 for reduction and modification, with the superfluous free PEI molecules then removed *via* filtration. Finally, the RGO/PEI composite powder is re-dispersed in 60 °C deionized water by ultrasonication and continuous stirring to obtain solution 3. Whatever the proportions of RGO and PEI, the overall concentration of the RGO/PEI composite in solution 3 is 0.5 wt% for all samples.

### 2.3 Preparation of the RGO/PEI composite films *via* layer-by-layer assembly

Using the solution 3 as precursor solution, a series of RGO/PEI composite films were prepared on PET substrates (10 × 10 cm) *via* a layer-by-layer assembly technique.^52,53^ Briefly, 1 mL of solution 3 was first coated on a corona-treated PET substrate using a glass coater and fully dried at 60 °C. This deposition cycle was repeated 20 times for each specimen, as shown in Fig. S2.† Details of the film preparation can be found in the ESI.† Samples are denoted by the additive amount of PEI in the solution 1: RGO/PEI-0.02, RGO/PEI-0.05, RGO/PEI-0.1, RGO/PEI-0.3, and RGO/PEI-0.5. It is important to remember that this additive amount is not the real proportion of PEI in the RGO/PEI composite films, because superfluous free PEI molecules have been removed during the filtration process, as mentioned above. In summary, the resulting RGO/PEI composite film is recasted, as a filtration and re-dispersion process was employed to prepare solution 3. The recast process was employed to realize both the sufficient reduction of GO and low content of PEI in the finally resulting films.

### 2.4 Characterization

In this study, measurements including scanning electron microscope (SEM), Fourier transform infrared spectroscopy (FTIR), X-ray diffraction (XRD), X-ray powder diffraction (XRPD), simultaneous thermal gravimetry (STG), gas (oxygen, nitrogen, and carbon dioxide) permeation, water vapor transmission rate (WVTR), and positron annihilation lifetime spectroscopy (PALS) were used. Details can be found in the ESI.†

## 3 Results and discussion

### 3.1 Reduction of GO and preparation of RGO/PEI composite films

**Fig. 2:**
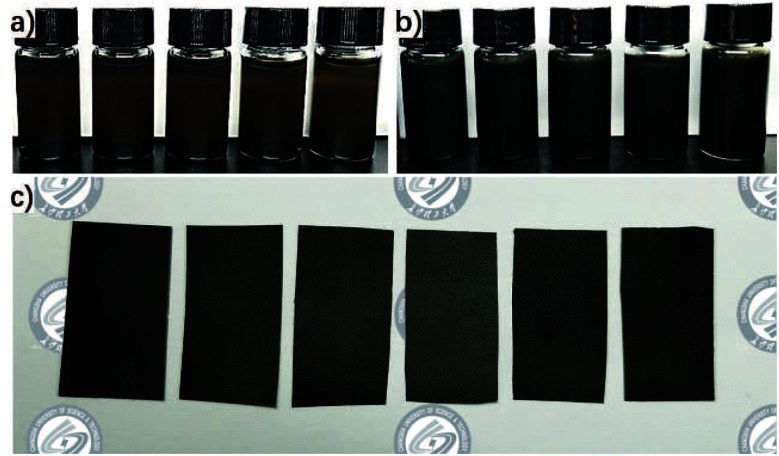
Pictures for (a) the solution 1, (b) the solution 2, and (c) the RGO/PEI composite films. The solutions are shown in the order of RGO/PEI-0.02, RGO/PEI-0.05, RGO/PEI-0.1, RGO/PEI-0.3, and RGO/PEI-0.5. The films are shown in the order of pure GO, RGO/PEI-0.02, RGO/PEI-0.05, RGO/PEI-0.1, RGO/PEI-0.3, and RGO/PEI-0.5.

**Fig. 3:**
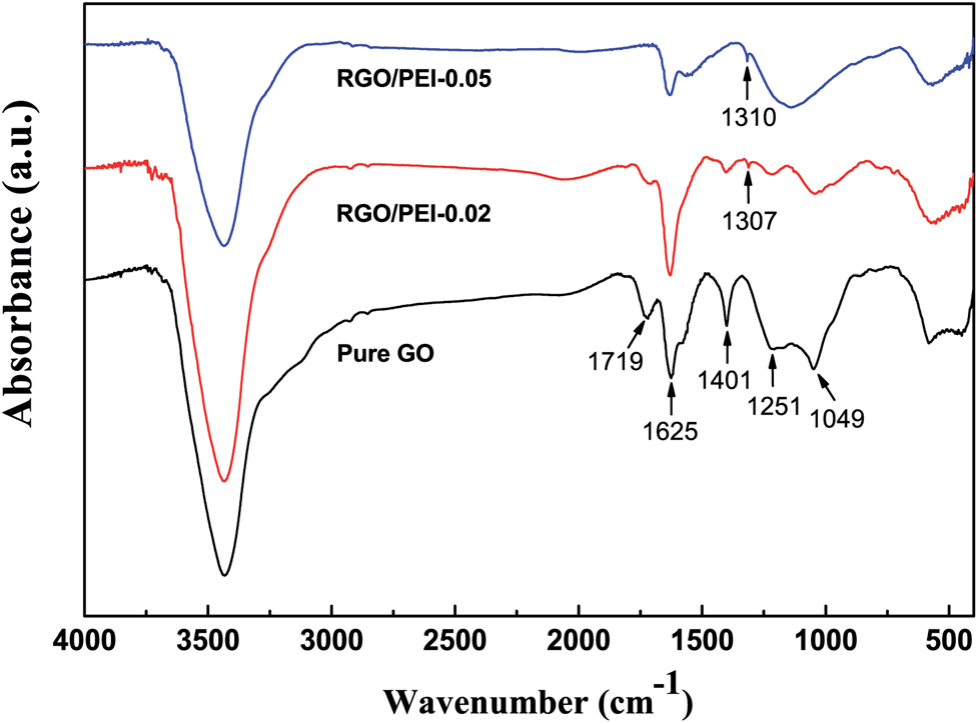
FTIR spectra of GO, RGO/PEI-0.02, and RGO/PEI-0.05 powder.

Fig. 2 includes pictures of solution 1, solution 2, and the resulting RGO/PEI composite films. There is an obvious change as solution 1 is yellow-brown while solution 2 is black, suggesting the transformation of GO into RGO. This is in good agreement with previous work.^17^ FTIR spectra of GO, RGO/PEI-0.02, and RGO/PEI-0.05 powders are shown in Fig. 3. In all spectra, the broad asymmetrical bands observed at 3200–3700 cm^−1^ are mainly due to water in the KBr powder, and the sharp peak at around 1625 cm^−1^ is from C–C in un-oxidized graphitic domains.^54^ Regarding the GO sheets, the absorption peaks at around 1251 cm^−1^ and 1049 cm^−1^ indicate the presence of epoxy C–O and alkoxy C–O, respectively. The peaks at 1401 cm^−1^ and 1719 cm^−1^ arise from the bending vibration of O–H groups and the stretching vibration of C=O, respectively. These peaks are in good agreement with previous work.^55^ Regarding RGO/PEI-0.05, the absence of peaks at 1049 cm^−1^, 1251 cm^−1^, 1401 cm^−1^, and 1719 cm^−1^ indicates the removal of epoxy groups, carboxylic groups, and hydroxyl groups, respectively. GO has evidently been reduced into RGO by PEI molecules in the RGO/PEI-0.05 composite.^56^ Further, the new peak appearing at 1310 cm^−1^ results from C–N, indicating that some PEI molecules have been successful grafted onto the RGO sheets. Regarding RGO/PEI-0.02, the intensity of peaks at 1049 cm^−1^, 1251 cm^−1^, 1401 cm^−1^, and 1719 cm^−1^ is greatly decreased. This suggests that the low concentration of PEI in the RGO/PEI-0.02 sample was not sufficient for the reduction of all GO sheets and a small amount of GO remains.

**Fig. 4:**
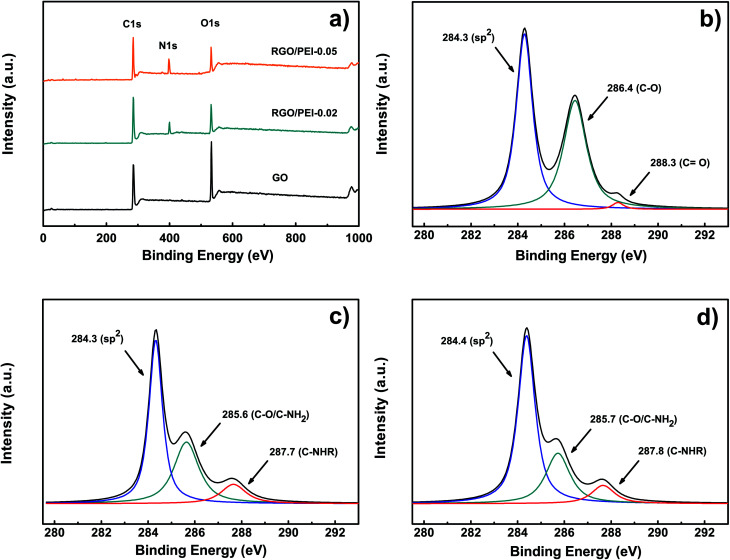
XPS (a) survey spectra of GO, RGO/PEI-0.02, and RGO/PEI-0.05, (b) C 1s of GO, (c) C 1s of RGO/PEI-0.02 and (d) C 1s of RGO/PEI-0.05.

A XPS measurement was used to analyze the chemical reduction and modification of GO by PEI molecules. Fig. 4a shows the survey spectra of GO, RGO/PEI-0.02, and RGO/PEI-0.05. For GO, the peaks observed at around 284 and 532 eV are attributed to C 1s and O 1s, respectively. The O 1s peak is significantly strong, which suggests a high oxidation degree of the GO. In the case of RGO/PEI-0.02 and RGO/PEI-0.05, a new N 1s peak at around 400 eV was found, indicating the introduction of PEI into RGO. Further, the intensity of the O 1s peaks are decreased, which suggests the reduction of GO by PEI. The C 1s peak of GO (Fig. 4b) can be deconvoluted into three components: the sp^2^ carbon bonds at 284.3 eV, the C–O bonds at 286.4 eV, and the C=O bonds at 288.3 eV. This result agrees well with that of the FTIR. For RGO/PEI-0.02 and RGO/PEI-0.05 (see Fig. 4c and d, respectively), the oxygenated species of C=O bonds are substantially removed, while the intensity of the C–O peak is dramatically reduced. This result confirmed the reduction of GO by PEI. Further, a peak at 287.7 or 287.8 eV has been noticed, which is attributed to the C-NHR bonds, caused by a nucleophilic reaction between amine and epoxy groups. This result confirmed the formation of covalent bonds between PEI and RGO. As a conclusion, in the resulting RGO/PEI composite films, GO has been successfully reduced into RGO and some PEI molecules were grafted onto it. The reduction mechanism of GO by PEI can be found in the ESI,† which is referenced from a reported work by Hongyu Liu *et al.*^17^

**Fig. 5:**
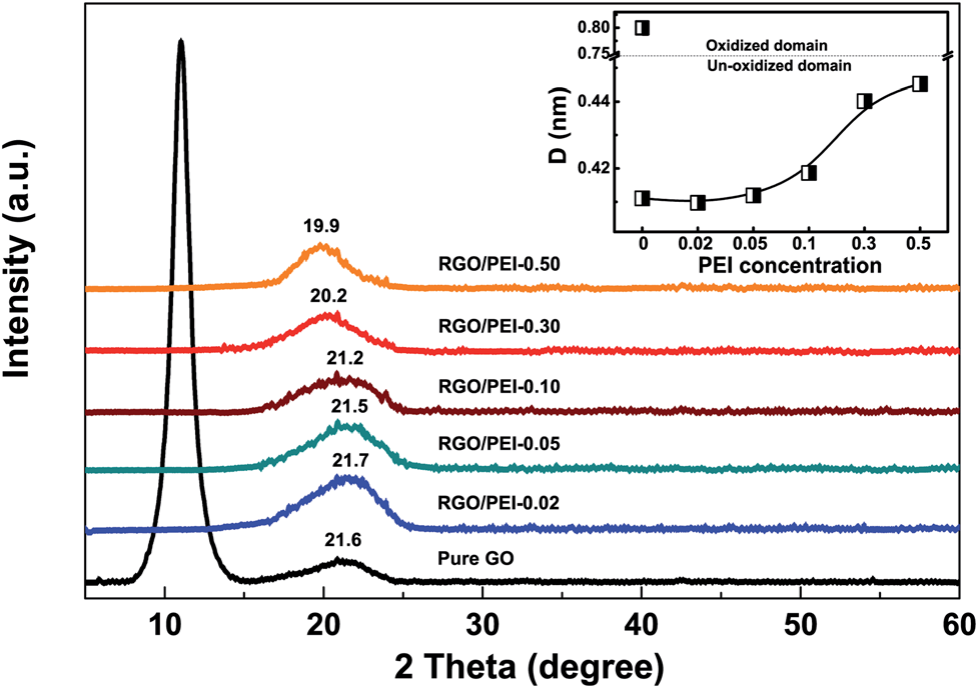
XRD patterns of GO and RGO/PEI composite powder. The insert shows the interlayer spacing of GO or RGO derived from the Bragg equation.

Fig. 5 shows the XRD patterns for pure GO film and RGO/PEI composite films on silicon wafers. For the pure GO film, a sharp peak at 2*θ* = 11.0° is observed. According to the Bragg equation,^57^ this peak indicates an interlayer spacing of *d* = 0.80 nm, a typical value for GO.^58^ An additional, less intense and broad peak can be seen at 2*θ* = 21.2°, corresponding to an interlayer spacing of *d* = 0.42 nm, a typical value for graphene.^59^ Thus, the XRD pattern characterized both the oxidized and the un-oxidized graphite domains in the GO film. For all RGO/PEI composite films, the peak at 11.0° disappeared. This result confirms that the PEI acts as an efficient reducing agent and the GO has been completely (or at least largely) reduced into RGO. The residual GO in the RGO/PEI-0.02 composite was not detected by XRD measurement, which may be attributed to its limited quantity or distribution as a monolayer. Further, at around 20°–22°, a broad peak can be noticed for all RGO/PEI composite films, and its diffraction angle downshifts with increasing the PEI concentration. This downshift in diffraction angles indicates an increment in interlayer spacing,^57,60^ which is likely attributed to the presence of PEI molecules.

### 3.2 Brick-wall structure of the RGO/PEI composite films

**Fig. 6:**
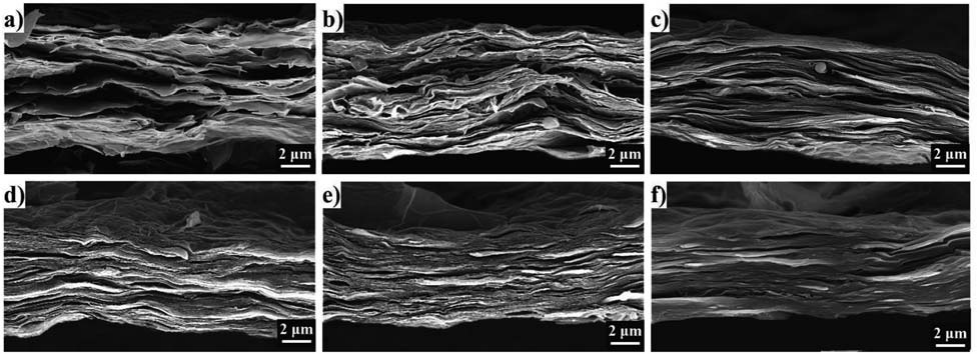
SEM images of the cross-section morphology of (a) pure GO film, (b) RGO/PEI-0.02, (c) RGO/PEI-0.05, (d) RGO/PEI-0.1, (e) RGO/PEI-0.3, and (f) RGO/PEI-0.5 composite films.

The cross-section morphologies of pure GO and RGO/PEI composite films on PS substrates are shown in Fig. 6. Films were freeze-fractured in liquid nitrogen to protect the cross-section. Both pure GO and RGO/PEI composite films show a clear multilayer structure, with the vast majority of GO and RGO sheets aligned parallel to the film surface. This observation confirms that layer-by-layer assembly is a very effective method for the preparation of highly ordered films. In the pure GO film, the delaminated structure is loose and porous, and many vacancies can be observed. As the PEI concentration is increased from 0.02 mg mL^−1^ to 0.5 mg mL^−1^, the delaminated structure of the film becomes more and more tight. In particular, RGO/PEI-0.05, RGO/PEI-0.1, RGO/PEI-0.3, and RGO/PEI-0.5 composite films show a homogeneous brick-wall structure, incorporating PEI as mortar and RGO platelets as nanobricks. The more PEI molecules the films possess, the fewer the vacancies observed in the brick-wall structure. The PEI molecules, physisorbed or covalently bonded on the surface of RGO sheets, entangle with each other or anchor along with the RGO sheets during the self-assembly procedure. With the graphene sheets dispersed in the PEI matrix uniformly, a continuous phase has been formed. In this case, the PEI molecules can be looked as a mortar component, while the RGO sheets act as bricks. This compact phase has excellent mechanical properties,^17–19^ making the RGO/PEI composite films suitable for barrier applications.

**Fig. 7:**
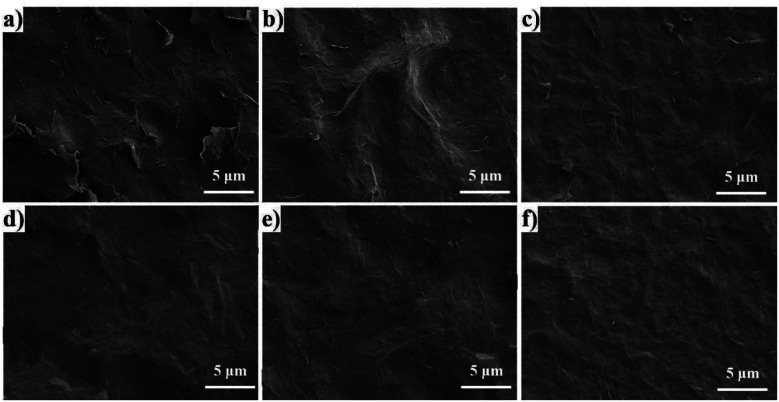
SEM images of the surface morphology of (a) pure GO film on PET substrate, (b) RGO/PEI-0.02, (c) RGO/PEI-0.05, (d) RGO/PEI-0.1, (e) RGO/PEI-0.3, and (f) RGO/PEI-0.5 composite films on PET substrate.

The surface morphologies of pure GO and RGO/PEI composite films are shown in Fig. 7. In the pure GO film (Fig. 7a), most GO sheets are overlapped with each other, and many cracks exist. Without PEI molecules, the interactions between GO sheets are insufficient to form a continuous and tight phase over a relatively long range. In the RGO/PEI-0.02 composite film (Fig. 7b), there are no cracks, and the film surface is basically continuous. However, some bulges occurred randomly on the film surface, resulting from the porous structure of the film. It is likely that vacancies occur under the bulges. The other RGO/PEI composite films all show largely flat and continuous surfaces, with the RGO sheets dispersed homogeneously in the PEI matrix. Therefore, higher PEI content is in favor of the dense structure of the RGO/PEI composite films, and the minimum concentration of PEI in solution 1 required to form a continuous and tight brick-wall structure is 0.05 mg mL^−1^.

Table 1 displays the thickness (*T*), the surface mass density (*ρ*_S_), and the mass density (*ρ*_M_) of the RGO/PEI composite films. For each specimen, the film thickness *T* was taken as the average of 10 points (based on the cross-section morphology measured by SEM) distributed over the sample. Surface mass density *ρ*_S_ was obtained according to the following equation,1
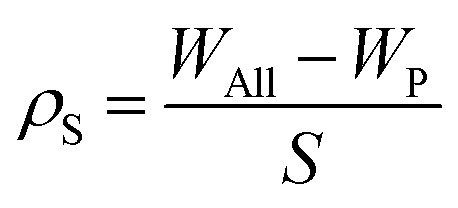
where *W*_P_ is the weight of the substrate. After the RGO/PEI composite film has been coated on the substrate, the sample was re-weighted and this value was used as the *W*_all_. S is the surface area of the substrate, which is 10 cm × 10 cm in this study. The *ρ*_M_ is obtained from the *ρ*_S_ and the film thickness *T*,2
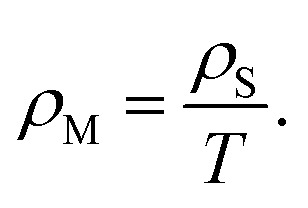


**Table 1:** Average thickness (*T*), surface mass density (*ρ*_S_), and mass density (*ρ*_M_) of the RGO/PEI composite films

Label	*T* (μm)	*ρ*_S_ (10^−3^ g cm^−2^)	*ρ*_M_ (g cm^−3^)
Pure GO	7.8	0.98	1.26
RGO/PEI-0.02	7.2	1.02	1.41
RGO/PEI-0.05	6.8	1.01	1.49
RGO/PEI-0.1	6.5	1.00	1.54
RGO/PEI-0.3	5.9	1.03	1.74
RGO/PEI-0.5	5.9	1.04	1.76

As shown in Table 1, the *ρ*_S_ values of all films are essentially the same, as they were all prepared using 20 mL of the corresponding solution 3 (0.5 wt%). Thus, there is little difference in total mass of the RGO/PEI composite films. As the PEI concentration was increased, the film thickness decreased (from 7.8 μm to 5.9 μm) because the film mass density *ρ*_M_ increased (from 1.26 g cm^−3^ to 1.76 g cm^−3^). This increase in density resulted from the PEI molecules, which bind to the surfaces of RGO and form a tight nanostructure. This increase in *ρ*_M_ indicates a denser structure, which should contribute to the gas barrier properties of the films (as discussed later in this study). In conclusion, these results demonstrate that the PEI molecules contribute significantly to the formation of a tight brick-wall structure in RGO/PEI composite films.

### 3.3 Component ratio of RGO/PEI composite films

**Fig. 8:**
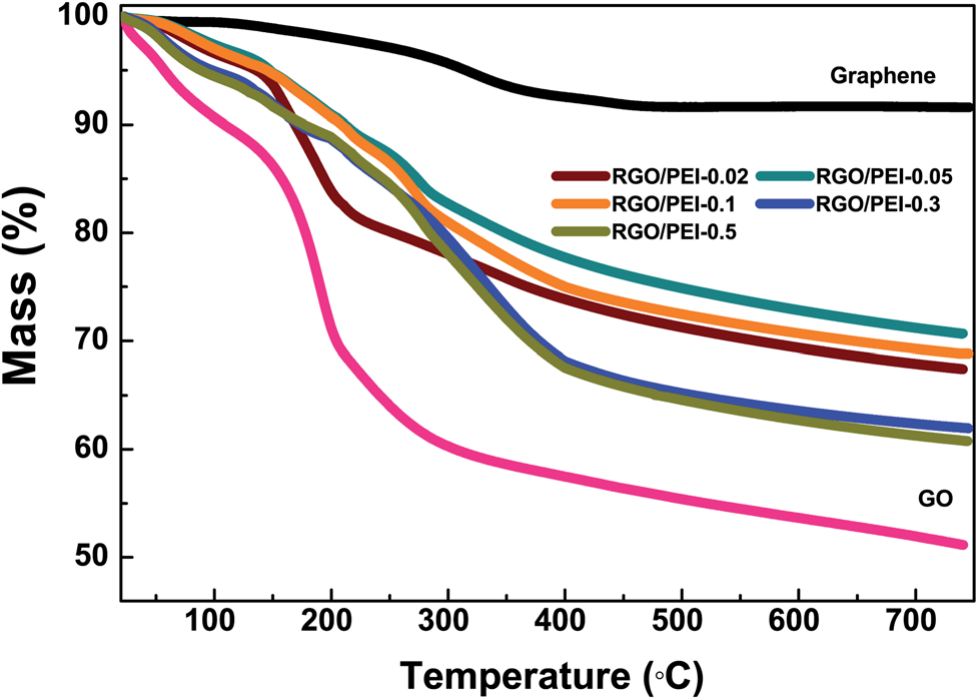
Thermal gravity analysis for the GO, the commercial graphene, and the RGO/PEI composite films.

To characterize the component ratios of RGO/PEI composite films, TG measurements have been performed as shown in Fig. 8. GO was found to be hydrophilic and unstable at elevated temperatures, a result of the abundant oxygen-containing groups on it. As temperature is increased from room temperature (RT) to 150 °C, the weight loss (13.8 wt%) is mostly due to the evaporation of water molecules. As the temperature is increased to 210 °C, another sharp weight loss (17.3 wt%) is observed, resulting from the pyrolysis of oxygen-containing groups.^61^ In comparison to GO, commercial graphene shows much better thermostability. Up to the highest temperature of the TG measurement, its weight loss is less than 9 wt%. Weight loss caused by the evaporation of water is also observed below a temperature of 150 °C in all RGO/PEI composite films, as the hydrophilic PEI molecules absorb water molecules in the presence of moisture. As the temperature is increased to 210 °C, only the RGO/PEI-0.02 composite film exhibits evident weight loss. This weight loss is mostly attributed to oxygen-containing groups in residual GO. Results confirmed that the GO has been reduced into RGO in the RGO/PEI-0.05, RGO/PEI-0.1, RGO/PEI-0.3, and RGO/PEI-0.5 composite films. Pyrolysis of PEI molecules generally occurs at temperatures ranging from 150 °C to 400 °C. Specifically, physisorbed PEI molecules mostly decompose at temperatures ranging from 150 °C–270 °C,^62^ while covalently bonded PEI molecules prefer to decompose at temperatures ranging from 270 °C–400 °C.^17,63^ In this case, except in RGO/PEI-0.02, the gradual weight loss experienced by samples in the temperature range 150 °C–270 °C can be largely ascribed to the pyrolysis of physisorbed PEI molecules, and that in the range from 270 °C–400 °C can be largely regarded as the pyrolysis of covalently bonded PEI molecules.

Based on the TG measurements, the amounts of water, oxygen-containing groups, physisorbed PEI molecules, covalently bonded PEI molecules, and total PEI molecules contained in the RGO/PEI composite films have been obtained and are summarized in Table 2. The water contents of the RGO/PEI composite films increase from 6.3 wt% to 8.4 wt% as a function of PEI concentration, while the water content of pure GO coating is 13.8 wt%. The significantly lower water contents of the RGO/PEI composite films is beneficial for their use in barrier applications. There are two reasons for the lower water content of these films. First, the hydrophilic GO has been reduced into hydrophobic RGO. Second, the superfluous free hydrophilic PEI molecules have been removed *via* the filtration process. The amount of physisorbed PEI molecules is basically the same for all RGO/PEI composite films (ranging from 9.3–10.7 wt%), because there is no significant difference in the van der Waals' force between PEI molecules and RGO sheets between samples. A clear increase in the amount of covalently bonded PEI molecules (from 5.5–14.6 wt%) has been found in films prepared with higher PEI concentration. Thus, the relatively high PEI concentration in solution 1 results in not only a more effective reduction of GO, but also a higher degree of surface modification of RGO by PEI. In addition, the comparatively high water content of the RGO/PEI-0.3 and RGO/PEI-0.5 composite films (8.1 wt% and 8.4 wt%, respectively) can be attributed to their relatively high content of PEI molecules (23.8.5 wt% and 24.1 wt%, respectively). In conclusion, the PEI molecules play a dominant role in the formation of RGO/PEI composite films by acting as both reducing agent and mortar.^17–19^ However, too much makes the film sensitive to external moisture.^36,37^

**Table 2:** The contents of water (*W*_w_), oxygen-containing groups (*W*_OCG_), physisorbed PEI molecules (*W*_phy-PEI_), covalently bonded PEI molecules (*W*_cov-PEI_), and total PEI molecules (*W*_all_) in the RGO/PEI composite films

Label	*W*_w_ (wt%) (RT–150 °C)	*W*_OCG_ (wt%) (150–210 °C)	*W*_Phy-PEI_ (wt%) (150–270 °C)	*W*_Cov-PEI_ (wt%) (270–400 °C)	*W*_All_ (wt%) (150–400 °C)
Pure GO	13.8	17.3	—	—	—
RGO/PEI-0.02	6.3	Unknown	Unknown	5.5	>5.5
RGO/PEI-0.05	5.2	—	9.4	7.7	17.1
RGO/PEI-0.1	5.3	—	10.7	9.0	19.7
RGO/PEI-0.3	8.1	—	9.3	14.5	23.8
RGO/PEI-0.5	8.4	—	9.5	14.6	24.1

Furthermore, it should be noted that, while the data in Table 2 was obtained according to a rigorous analysis, the resulting data may differ slightly from the actual values, as approximations were made. For example, the weight loss in the temperature range 150 °C–210 °C is not entirely caused by the decomposition of oxygen-containing groups. Moreover, although 270 °C was used as the cut-off point for the decomposition temperature for the physisorbed PEI and the covalently bonded PEI, the real cut-off point is ambiguous; it is not a sharp discontinuity in reality. Thus, the data in Table 2 are approximate. Nevertheless, this data is basically reliable, and can be used to study the component ratios of the films. In addition, the *W*_OCG_ and *W*_phy-PEI_ values for the RGO/PEI-0.02 composite film are not shown in Table 2, because both the oxygen-containing group and the physisorbed PEI molecules decompose at the temperature range 150 °C–210 °C and therefore cannot be distinguished.

### 3.4 Gas-barrier properties of RGO/PEI composite films

**Fig. 9:**
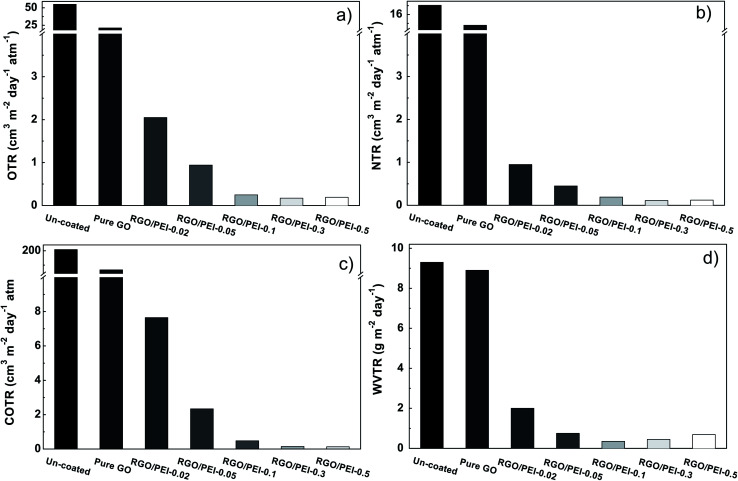
(a) Oxygen transmission rate (OTR), (b) nitrogen transmission rate (NTR), (c) carbon dioxide transmission rate (COTR), and (d) water vapor transmission rate (WVTR) for the RGO/PEI composite films on PET substrate.

Fig. 9 shows the oxygen transmission rate (OTR), nitrogen transmission rate (NTR), carbon dioxide transmission rate (COTR), and water vapor transmission rate (WVTR) of the RGO/PEI composite films on a PET substrate. Clearly, even without the PEI additives, the 7.8 μm pure GO coating contributes to the oxygen, nitrogen, and carbon dioxide barrier property, despite the porous nature of GO sheets and the loose structure of the film, which indicates a large gallery spacing. A significant reduction in OTR, NTR, and COTR has been found for all RGO/PEI composite films as a function of PEI concentration. Unlike with the pure GO coating, the RGO sheets in the RGO/PEI composite films are tightly stacked in a brick-and-mortar structure. Both the physisorbed PEI and the covalently bonded PEI molecules interact with the RGO sheets and form a compact composite. The large gallery spacing between the RGO sheets, and any defects in the RGO sheets, can be filled and blocked by these PEI molecules. As described by Cussler's model,^12,13^ during permeation, gas molecules must avoid the RGO sheets (acting as impermeable inorganic filler) and tend to wiggle through the permeable polymer channels until they find a slit between RGO sheets to penetrate into the next channel. This phenomenon leads to a higher tortuosity of the permeation path.^64^

As shown in Table 3, the low oxygen, nitrogen, and carbon dioxide permeability of RGO/PEI confirms that the RGO/PEI coating contributes significantly to the gas barrier property. These permeability values are fairly good in comparison with previous studies.^9–27^ As shown in Table S1,† the RGO/PEI composite films studied in this work show relatively low oxygen gas permeability when compared with various gas barrier films prepared by the layer-by-layer method. In particular, the RGO/PEI-0.3 coating (23.8 wt% PEI content) shows minimum oxygen and nitrogen permeabilities of 0.116 and 0.076 × 10^−21^ m^3^ m m^−2^ Pa^−1^ s^−1^, respectively. As the PEI content is increased from 23.8 wt% to 24.1 wt%, a slight increase in oxygen/nitrogen permeability has been found, possibly due to the plasticization of film with higher water content (see Table 2). The RGO/PEI-0.5 coating (24.1 wt% PEI content) shows a minimum carbon dioxide permeability of 0.089 × 10^−21^ m^3^ m m^−2^ Pa^−1^ s^−1^. This is due to the strong interaction between the acid gas molecules of carbon dioxide and the amino groups on PEI. After having dissolved in the alkaline water, carbon dioxide is difficult to dissolve out. Thus, the more PEI in the RGO/PEI composite film, the better it functions as a carbon dioxide barrier.

**Table 3:** The oxygen, nitrogen, and carbon dioxide permeability of RGO/PEI composite films. Numbers in the brackets have a certain error, resulting from the detectable limit of the instrument

Label	Overall/coating (10^−21^ m^3^ m m^−2^ Pa^−1^ s^−1^)		
	O_2_ permeability	N_2_ permeability	CO_2_ permeability
Un-coated	222.23(4)/—	81.54(5)/—	826.99 (7)/—
Pure GO	105.11(8)/31.24(1)	55.18(4)/22.51(9)	661.27(0)/348.18(1)
RGO/PEI-0.02	10.01(3)/1.77(5)	4.64(0)/0.83(1)	37.36(5)/6.62(3)
RGO/PEI-0.05	4.54(8)/0.75(3)	2.17(7)/0.36(2)	11.32(1)/1.86(3)
RGO/PEI-0.1	1.20(1)/0.18(9)	0.91(3)/0.14(4)	2.30(6)/0.36(2)
RGO/PEI-0.3	0.80(5)/0.11(6)	0.52(1)/0.07(6)	0.71(0)/0.10(3)
RGO/PEI-0.5	0.89(9)/0.13(0)	0.56(8)/0.08(2)	0.61(5)/0.08(9)

The permeability of RGO/PEI composite films (*P*_all_) is determined by that of PET substrate (*P*_P_) and RGO/PEI coating (*P*_C_), as follows:^65,66^3
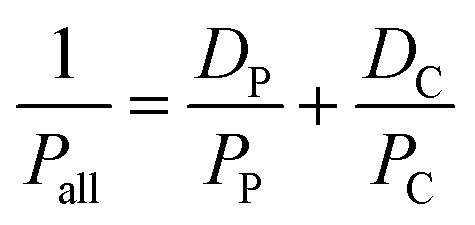
where *D*_P_ is the volume fraction of PET substrate in the whole films, and *D*_C_ is that of RGO/PEI coating in the whole films, as follows,4
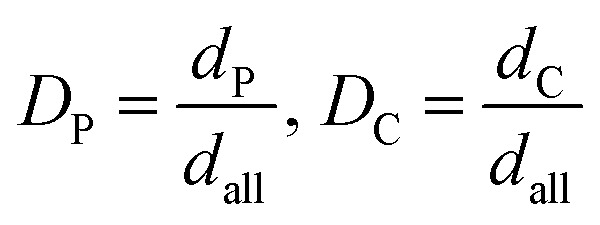
where *d*_P_, *d*_C_, and *d*_all_ are the thicknesses of PET substrate, RGO/PEI coating, and overall film, respectively.

The WVTR was measured by a cup method^67^ (as described in the ESI†), under a constant environment, with 80% relative humidity at 25 °C. This measurement was extended to a relatively long period (15 days), to determine the long-term water vapor barrier effectiveness of the films. As expected, a layer of pure GO shows minimal influence on the WVTR. The hydrophilic GO sheets, which absorb moisture from the air, make the GO coating water-rich (13.8 wt%, see Table 2). Thus, the permeation pressure of water vapor on the two sides of the PET substrate increased, which increased the WVTR of the films. In contrast, the RGO/PEI composite films all show excellent water vapor barrier properties. In particular, the WVTR of the RGO/PEI-0.1 composite film was 0.35 g m^−2^ day^−1^, a reduction of 96% compared with un-coated PET film (9.3 g m^−2^ day^−1^). This significantly enhanced water vapor barrier property is primarily the result of two factors. First, the brick-wall multilayer structure, as described above. Second, the hydrophobic RGO sheets reduce the water content of the films as well as the permeation pressure of water vapor on the two sides of the PET substrate.

As the PEI content is increased from 19.7 wt% to 24.1 wt%, the WVTR of the films gradually increased. As shown in Table 2, this increase in WVTR is accompanied by an increase in water content, due to the presence of additional hydrophilic PEI molecules. Thus, a PEI concentration of around 0.1 mg mL^−1^ in the precursor solution (19.7 wt% in the film) creates an optimized barrier for water vapor. Less than this value, there are not enough PEI molecules to realize complete reduction of GO, bind the surfaces of RGO, create a tight nanostructure, and fill and block the gallery spacing between the RGO sheets and the defects in the RGO sheets. Above this value, the superfluous PEI molecules attract too much water into the RGO/PEI composite films, resulting in plasticization and increasing the gallery spacing.^36,37^ In conclusion, to form barriers against water vapor, nitrogen/oxygen, and carbon dioxide, the optimal content of PEI in the composite film has been found to be 19.7, 23.8, and 24.1 wt%, respectively. These are relatively low values compared with previously reported studies, which indicates that 24.1 wt% PEI molecules are enough for the formation of a brick and mortar structure with high barrier efficiency.

Further, the influence of the layer number on the OTR and WVTR of RGO/PEI-0.3 and RGO/PEI-0.5 composite films has been investigated, and the results are shown in Fig. S3 and S4.† Increasing the number of layers from 0 to 5 sharply decreases the OTR from 54.86 to 5.13 and 6.56 cm^3^ m^−2^ day^−1^ atm^−1^ for RGO/PEI-0.3 and RGO/PEI-0.5 composite films, respectively. Further increasing the number of layers results in a gradual decline in OTR. Thus, a few number of RGO/PEI layers are effective for the oxygen barrier property. Whereas, as shown in Fig. S4,† the WVTR shows a relatively uniform decrement as a function of the number of layers, which indicates that the length of diffusion pathway plays a decisive role for the barrier for water molecules.

### 3.5 Permeation of gas molecules within RGO/PEI composite films in terms of free volume

**Fig. 10:**
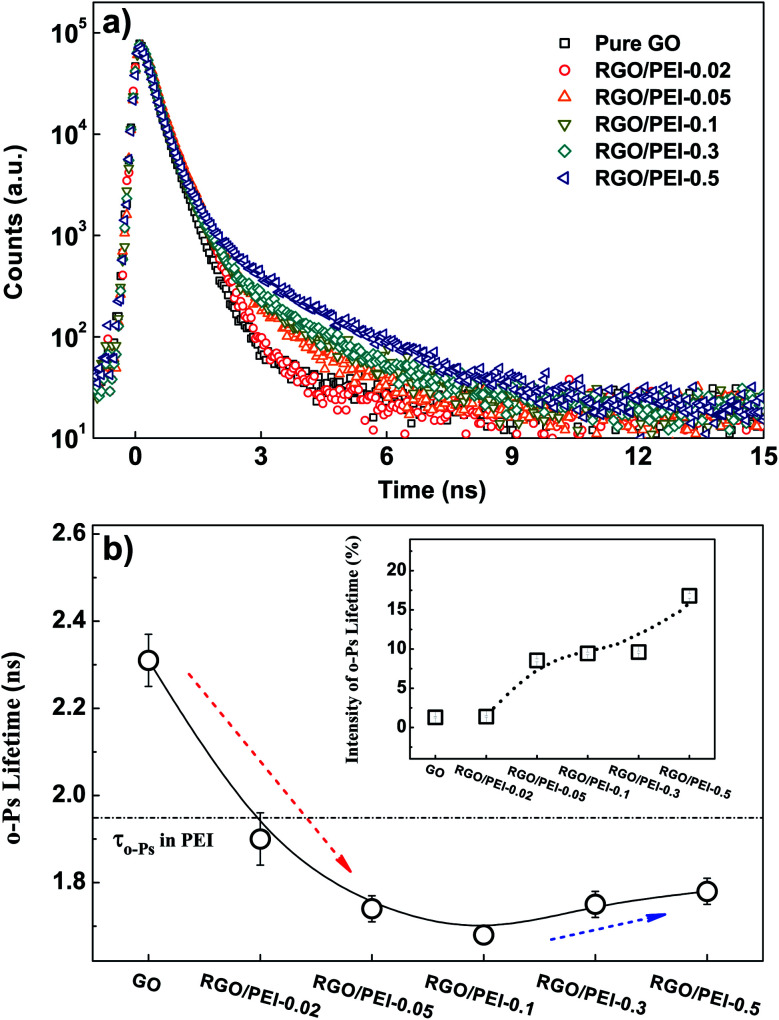
(a) The raw positron lifetime spectra of samples; (b) the variations in o-Ps lifetime (*τ*_o-Ps_) and the intensity (*I*_o-Ps_) in the PEI/RGO composite films. Dotted arrows are guides to the eyes. Dotted line marks the o-Ps lifetime for pure PEI.

The free volume theory posits that gas molecules in polymers can migrate only when surrounded by large enough free volume holes.^68–79^ Thus, it is of primary importance to analyze the gas permeation within the free volume in PEI/RGO composite films. After all other measurements had been completed, the PEI/RGO composite films were carefully scraped off the substrate, and the resulting powder-like PEI/RGO composites were mechanically pressed to discs for PALS measurement. Details about the mechanism of PALS measurement and the calculation of free volume size according to the o-Ps lifetime can be found in the ESI.† Measurement was conducted at 25 °C and ambient room humidity. The raw positron annihilation lifetime spectra for all samples are shown in Fig. 10a.

The PALS analysis program PASA and LT^80,81^ were applied to analyze the positron lifetime spectra. The variances of the fits were in the range of 0.97–1.20. Only one long-lived o-Ps lifetime (*τ*_o-Ps_) can be derived from the spectra for all samples, indicating there is a single type of free volume hole among them. Both *τ*_o-Ps_ and the intensity (*I*_o-Ps_) are displayed in Fig. 10b. By using the spherical approximation given by the Tao–Eldrup model,^82,83^ the average size of hole free volumes can be calculated according to the value of *τ*_o-Ps_; the results are shown in Table 4. The free volume holes in GO are apparently large (126.83 × 10^−3^ nm^3^) and sparse (*I*_o-Ps_ = 1.30%), indicative of the loose structure of GO. Nano-enclosed spaces in it are both large in size and small in amount. As the PEI content increased from 0 wt% to ∼19.7 wt% (Table 2), the free volume size decreased from 126.83 × 10^−3^ nm^3^ to a minimum value of 68.27 × 10^−3^ nm^3^ (red dotted arrow). This decrease in free volume size can be attributed to a synergistic effect. First, there is the binding effect of PEI molecules on the RGO sheets, which not only facilitates the formation of a tight brick-and-mortar structure, but also shrinks the free volume holes. Second, the long o-Ps lifetime in GO is no longer present, because GO has been reduced into RGO. The o-Ps can hardly be formed in the graphene sheets.^84^

**Table 4:** The o-Ps lifetime (*τ*_o-Ps_), the radius of free volumes (*R*_FV_), and the sizes of free volumes (*V*_FV_) obtained by using the Tao–Eldrup model

Label	o-Ps lifetime (ns)	*R*_FV_ (10^−1^ nm)	*V*_FV_ (10^−3^ nm^3^)
GO	2.31	3.12	126.83
RGO/PEI-0.02	1.90	2.76	87.61
RGO/PEI-0.05	1.74	2.60	73.41
RGO/PEI-0.1	1.68	2.54	68.27
RGO/PEI-0.3	1.75	2.61	74.28
RGO/PEI-0.5	1.78	2.64	76.90

As the free volume theory makes clear, the permeability of gas molecules in polymers is sensitive to the size, density, and distribution of free volumes, as well as the fraction free volume.^71–89^ Moreover, the size, solubility, and weight of the gas molecules also have a significant influence on their permeation.^44–46^ Thus, gas permeation in polymer is a very complex phenomenon, and there is rarely a simple linear relation between the gas permeability and a given parameter.^44–46,90^ We found that there is no linear relation between the free volumes and the gas permeability for the PEI/RGO composite films. Nevertheless, the PALS results strongly suggest that the decrease in free volume size has made a significant contribution to the excellent gas barrier property of the PEI/RGO composite films.

As the PEI content was further increased from 19.7 wt% to 24.1 wt%, *τ*_o-Ps_ increased slightly (blue dotted arrow). The measured value of *τ*_o-Ps_ in PEI is 1.95 ns (see Table 5). Thus, as more and more o-Ps are able to form and annihilate in PEI, with a longer lifetime, the average *τ*_o-Ps_ is slightly increased, despite the compact structure of the films. In addition, considering the very low value of *I*_o-Ps_ in pure GO, this abundant annihilation of o-Ps in PEI (*I*_o-Ps_ = 20.67%) is likely to also be responsible for the increase in *I*_o-Ps_ from 1.30% to 16.79% as a function of PEI content (see the insert of Fig. 10b).

**Table 5:** Lifetime and intensity components obtained from the PALS spectra of PEI

	Component 1	Component 2	Component 3
Lifetime *τ* (ns)	0.125	0.365	1.95
Intensity *I* (%)	4.93	74.40	20.67

## 4 Conclusions

In this study, a series of RGO/PEI composite films have been synthesized using recast and layer-by-layer deposition processes. The recast process ensures that GO sheets can sufficiently contact and react with myriad PEI molecules (the PEI: GO feeding ratio is 0.02 : 0.1, 0.05 : 0.1, 0.1 : 0.1, 0.3 : 0.1 and 0.5 : 0.1) in the precursor solution. Thus, the GO sheets are reduced into RGO with the PEI molecules acting as reducing agent, and some PEI molecules can be covalently bonded or physisorbed on the RGO. Finally, the superfluous free PEI molecules are removed by a filtration process. Results reveal that the content of PEI (covalently bonded and physisorbed) in the films ranges from >5.5 wt% to 24.1 wt%. This is a relatively low value compared with previously reported studies, which indicates that 24.1 wt% PEI molecules are enough for the formation of a homogeneous brick-wall multilayer structure with high barrier efficiency due to the strong interactions between PEI chains and RGO. In addition, the low content of hydrophilic PEI molecules avoid the film to adsorb too much water molecules, which causes plasticization and increases the gas permeability. These RGO/PEI composite films with a brick-wall multilayer structure show excellent gas barrier properties. In particular, the optimal proportion of PEI and RGO in the composite film for its gas barrier properties towards different gases has been determined, as follows: the RGO/PEI-0.1 composite film (19.7 wt% PEI) is the optimal barrier for water vapor (0.35 g m^−2^ day^−1^), the RGO/PEI-0.3 composite film (23.8 wt% PEI) shows the lowest permeability for oxygen/nitrogen (0.116/0.076 × 10^−21^ m^3^ m m^−2^ Pa^−1^ s^−1^), and the best barrier for carbon dioxide (0.089 × 10^−21^ m^3^ m m^−2^ Pa^−1^ s^−1^) occurs with the RGO/PEI-0.5 composite film (24.1 wt% PEI). According to the PALS measurements, the free volumes in the RGO/PEI composite films are small, one of the most important reasons for their excellent gas barrier properties. In summary, the mechanism for the excellent gas barrier property of the RGO/PEI composite films is a synergistic effect, resulting from the combination of their compact brick-wall structure, small free volumes, optimized component ratio, high density, and hydrophobicity.

## Conflicts of interest

There are no conflicts to declare.

## Supplementary Material

RA-012-D1RA09205G-s001
