# Supplementary material for: Gas permeation and microstructure of reduced graphene oxide/polyethyleneimine multilayer films created via recast and layer-by-layer deposition processes
Source: RSC Adv. 2022 Feb 25;12(11):6561–72. doi: 10.1039/d1ra09205g (PMC8982006; doi:10.1039/d1ra09205g)
Supplement: RA-012-D1RA09205G-s001 [file RA-012-D1RA09205G-s001.pdf]

Electronic Supplementary Material (ESI) for RSC Advances.
This journal is © The Royal Society of Chemistry 2022

Supporting Information

Gas permeation and microstructure of reduced graphene oxide/polyethyleneimine multilayer films created via recast and layer-by-layer deposition processes

Chongshan Yin[a*], Xuan Du[a], Zhi Ding[a], Qing Zeng[a], Xi Li[a], Chunqing He[b*], Bangyun Xiong[c], Jingjing Li[c], Yawei Zhou[d]

[a] Hunan Provincial Key Laboratory of Flexible Electronic Materials Genome Engineering, School of Physics and Electronic Science, Changsha University of Science and Technology, Changsha 410114, China.

[b] Key Laboratory of Nuclear Solid State Physics Hubei Province, School of Physics and Technology, Wuhan University, Wuhan 430072, China.

[c] School of Materials Science and Energy Engineering, Foshan University, Foshan 528000, China.

[d] State Key Laboratory of Intense Pulsed Radiation Simulation and Effect, Northwest Institute of Nuclear Technology, Xi'an 710024, China.

*Corresponding Author. E-mail address: c.sh.yin@foxmail.com.

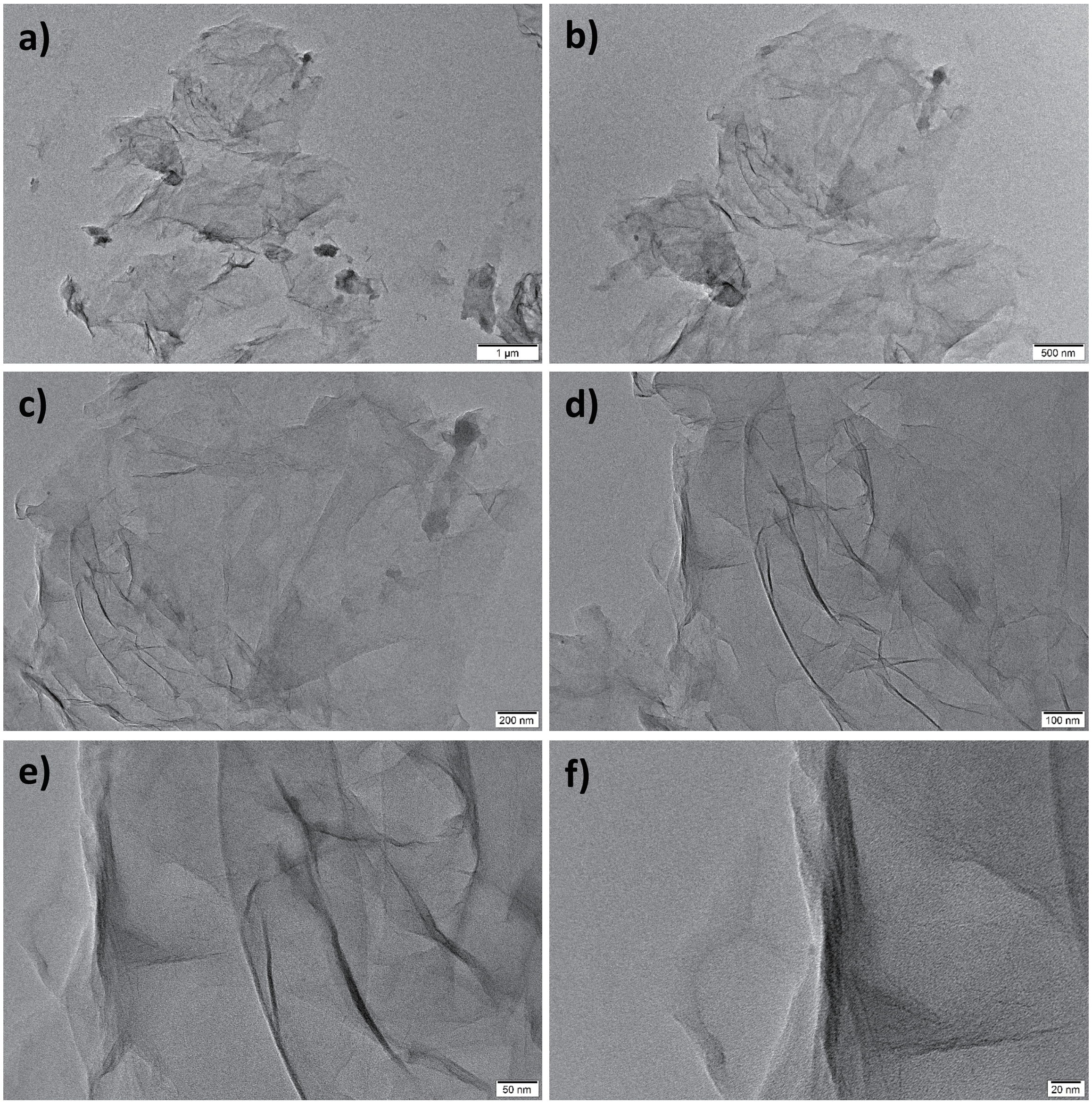

Figure S 1 Schematic illustration of the preparation of a RGO/PEI composite film by using layer-by-layer assembly.

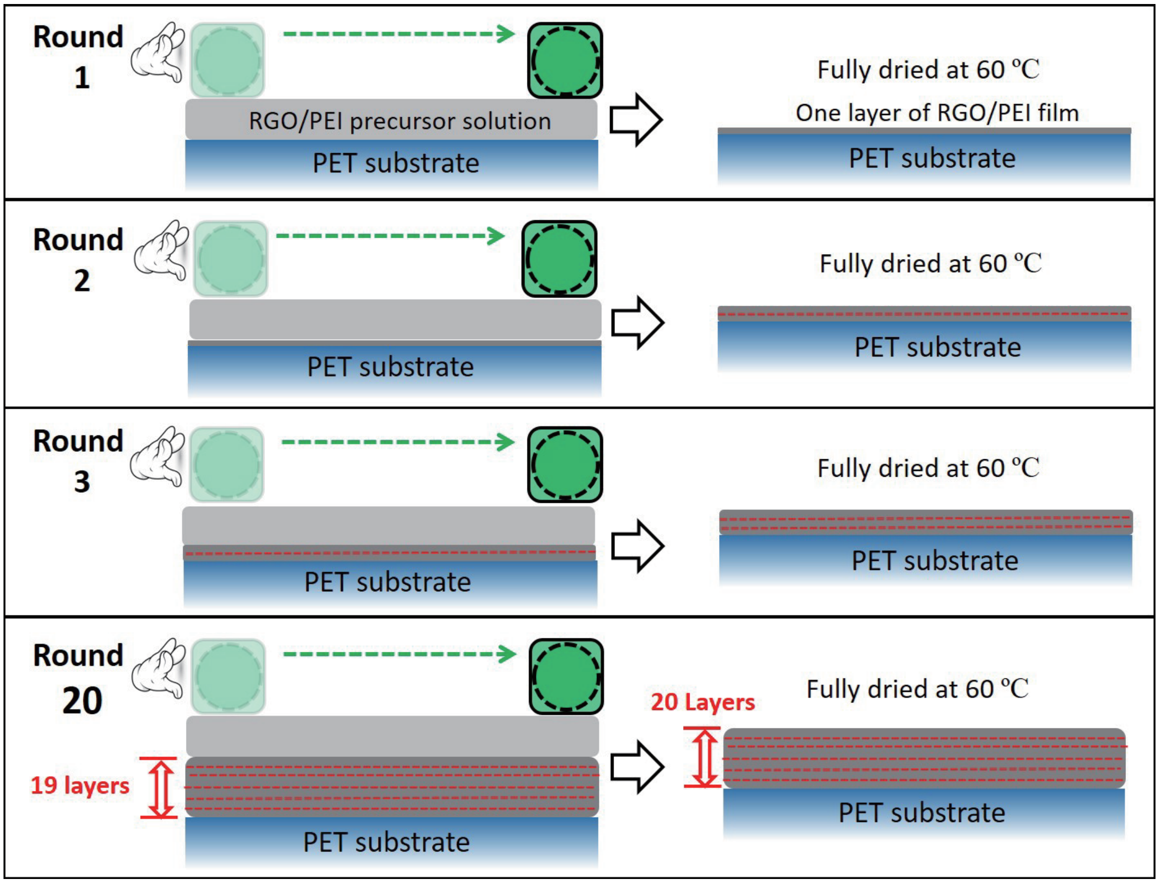

Figure S 2 TEM images of the GO sheets with different magnification.

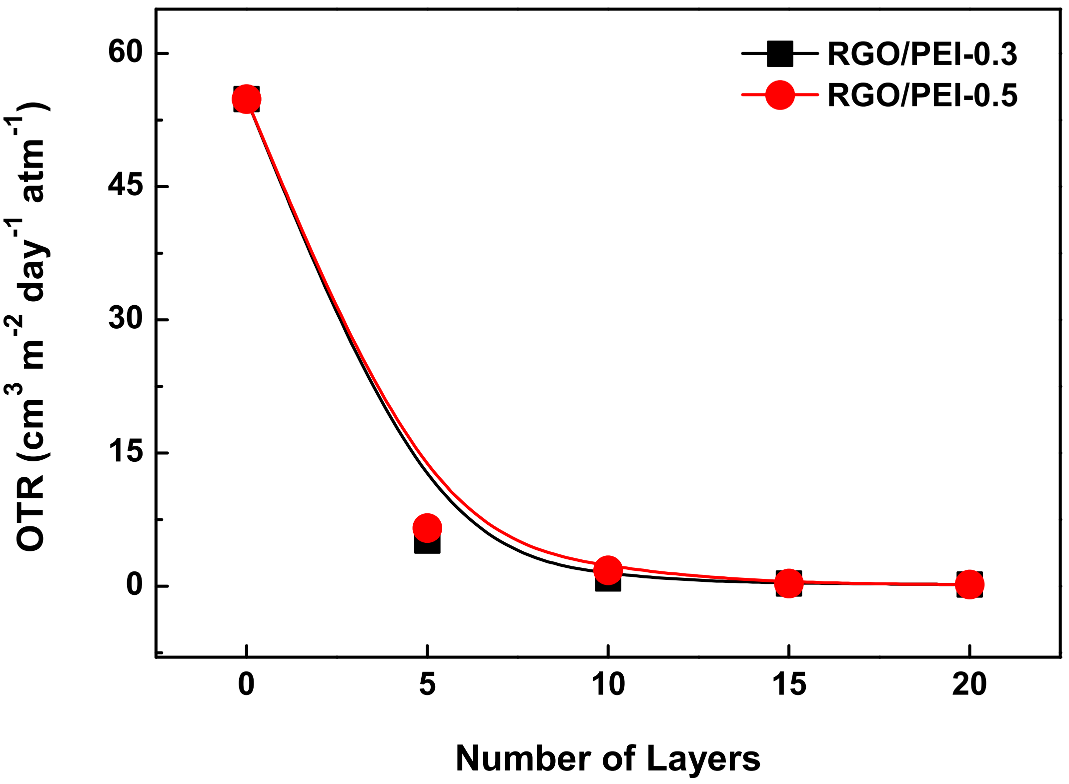

Figure S 3 Oxygen transmission rate (OTR) of the RGO/PEI-0.3 and RGO/PEI-0.5 composite films on PET substrate.

Table S1. Survey of oxygen permeability for various gas barrier films prepared by a layer-by-layer method.

Polymer matrix	*Filler*	*Film composition*	*Oxygen permeability* ($10^{-21} m^3 m\ m^{-2} Pa^{-1} s^{-1}$)	*Ref*
Charged polyurethane	*GO*	$(PU+/GO/PU-)_{60}$	1.01	[1]
PEO/PAA	*MMT*	$(PEO/PAA+MMT)_{10}$	0.2	[2]
Polyglycidol/PEI	*MMT*	$(PGD/PEI/MMT)_{20}$	0.04	[3]
PAA/PEO	–	$(PAA/PEO)_{20}$	4.10	[4]
PEI/PAA/PEO	–	$(PEI/PAA/PEO/PAA)_{20}$	1.11	[5]
TA/PEO	–	$(TA/PEO)_{40}$	6.66	[6]
PVA	*RGO*	$(PVA/rGO_{0.5})_5$	0.77	[7]
PEI	*RGO*	$(PEI/RGO)_{20}$	0.116	*This work*

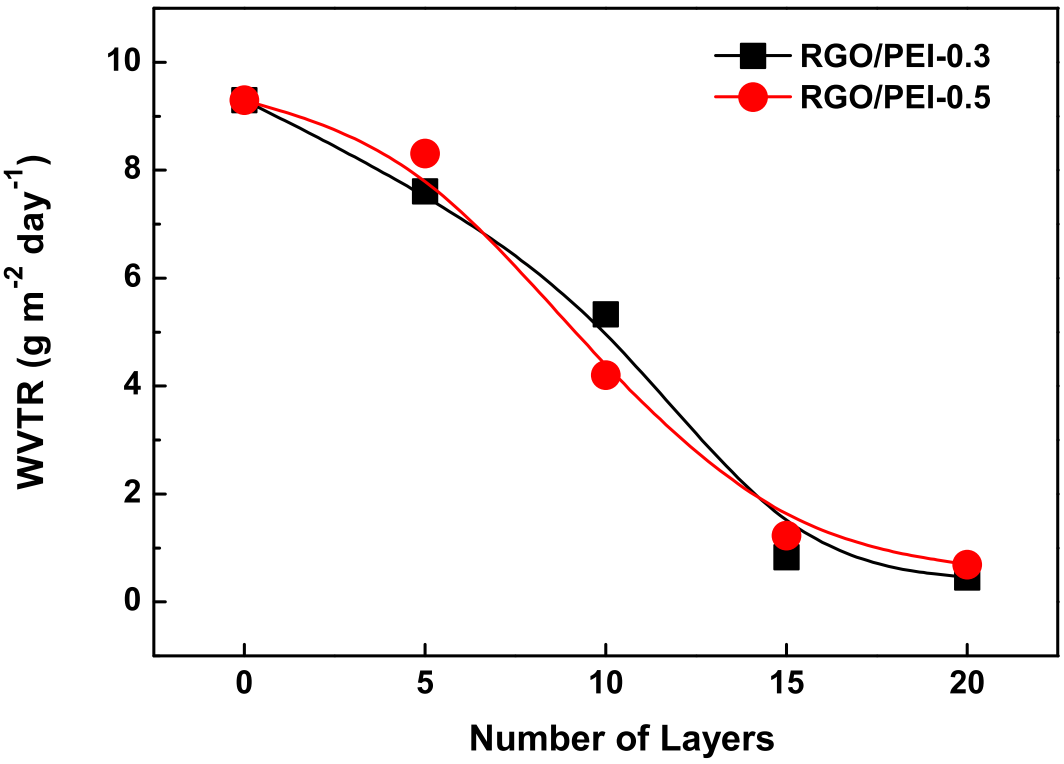

Figure S 4 Water vapor transmission rate (WVTR) of the RGO/PEI-0.3 and RGO/PEI-0.5 composite films on PET substrate.

Layer-by-layer assembly technique for the preparation of RGO/PEI composite films.

A similar layer-by-layer assembly technique has been reported by the same research group [8, 9], and a schematic can be found in Figure S1:

Step 1: 20 mL of the Solution 3 (as mentioned in the Experimental of the manuscript) was used for each sample - just enough to produce one RGO/PEI composite film with a surface mass density of $\sim 1.0 \times 10^{-3}$ g cm^{-2} on a 10 cm $\times$ 10 cm PET substrate.

Step 2: 1 mL of the Solution 3 was carefully coated on a PET substrate using a glass coater. The suspension was fully evaporated at 60 °C.

Step 3: Step 2 was repeated 20 times to prepare a film consisting of 20 layers of RGO/PEI composite.

The mechanism of the layer-by-layer assembly as well as the parallel arrangement of RGO sheets is that: For each layer, only a small amount of precursor solution was used, which is just enough to prepare one ultrathin layer of RGO/PEI composite (< 0.4 μm). When dispersed in an ultrathin layer, the RGO sheets can be forced to align parallel to the layer due to the space limitation. With the PEI molecules spread laterally in the layer with a high tendency, the strong interaction between PEI chains and RGO sheets ensures the formation of the RGO/PEI composite film with homogeneous brick-wall structure [9–11]. Similar technique has been reported by the same research group [9].

The reduction mechanism of GO by PEI.

The reduction mechanism of GO by PEI is shown here, which is referenced from a reported work by Hongyu Liu et.al [11]. The epoxy group can be reduced by the amine groups with

a nucleophilic ring-opening reaction, followed by an elimination reaction. Another possible approach to reduce epoxy groups by amine groups was that the -NH_2 functionalities of PEI may attack the epoxide carbon of GO to convert it into a hydroxyl group accompanying a proton transfer. Subsequently, an intermediate is formed by releasing water molecules from the surface of GO because of the high binding affinity of protons with the hydroxyl groups. The proton from the methylene carbon can be removed and the intermediate structure is transformed into C=C in the GO. The two adjacent hydroxyl groups first form the transition state of the epoxy group. Then, the resultant epoxy group is reduced by the amine group. Hydroxyl groups may also be eliminated together with the hydrogen attached with b-carbon to form C=C. In the case of carbonyl and carboxylic groups, the amine groups can react with the carbonyl group via a Michael addition/Schiff base reaction. The carboxylic groups are very labile, and this fact is responsible for the removal of carboxylic groups from the RGO sheets at a high temperature of 80 °C.

Fourier transform infrared spectroscopy (FTIR), simultaneous thermal gravimetry (STG) measurements, Scanning Electron Microscope (SEM), X-ray diffraction (XRD), and X-ray powder diffraction (XRPD).

FTIR was used to determine surface chemical bonding and structure of the GO and RGO sheets. Before measurements, samples were mixed with the dry KBr powder and were mechanically pressed to discs. FTIR was carried out at room temperature at a resolution of 2 cm^{-1} by a Nicolet 170 SXIR spectrometer in the range of 4000 cm^{-1} to 500 cm^{-1}. STG data were collected on a Netzsch STA449C at 5 °C min^{-1} under N_2 purge. The morphologies of RGO/PEI composite films were measured by the SEM (Sigma HD, Carl Zeiss Jena, Germany). The XRD and XRPD measurements were performed on a D8 ADVANCE type diffractometer, and the scan rate was 4°

min^{-1}.

Water vapor transmission rate (WVTR) and gas (nitrogen, oxygen, and carbon dioxide) permeability measurement

A cup method was used to obtain the WVTR of films [12]. First, fill a cup (made of hard plastics) with a certain amount of anhydrous calcium chloride ($CaCl_2$), and seal the cup with a tested film. The side with RGO/PEI composite films was in contact with air (laboratory environment, 80% relative humidity and 25 °C). Then, keep the cup under this constant laboratory environment, measuring and recording the mass of cup over a period of time. WVTR is defined as the Equation as shown below,

$$WVTR = \frac{\Delta m \cdot d}{A \cdot t} \tag{1}$$

where Δm is the mass difference between two consecutive measurements within a time of t, d is the thickness of the film, A is the effective area of the film used for water vapor transmission, and t is the time interval between two consecutive measurements.

Gas (Hydrogen, oxygen, carbon dioxide) permeability was measured by a differential pressure method on a Labthink VAC-V3 apparatus, monitoring the amounts of gas molecules that permeated across the membrane from one side to the other, under a constant environment condition (0% relative humidity and 25 °C). The operating pressure difference is 1 atm.

Positron annihilation lifetime measurements

The PEI/RGO composite were carefully scraped off the PET substrate, and the resulting powder-like PEI/RGO composites were mechanically pressed to discs. The positron annihilation experiments were conducted by using a fast-fast coincidence PALS with a time resolution function of 0.230 ns for the full width at half maximum (FWHM), and 1 million counts were collected

for each spectrum. A ^{22}Na source ($\sim$10 μCi) was firstly sandwiched by 2 PEI/RGO composite discs and then covered with an Al foil. The membranes with the source were placed in a testing chamber, at room temperature. The PATFIT program [13] as well as the LT program [14] were applied to analyse the positron lifetime spectra of the membranes, and the variances of the fits were in the range of 0.97$\sim$1.20. Details of PALS measurements set up can be found in our recent paper [15].

Application of positron annihilation lifetime spectroscopy (PALS) to polymer membranes relies on the fact that a part of the injected positrons combine with electrons from surrounding molecules to form positronium (Ps) atoms in them. Ps is a hydrogen-like bound state of a positron and an electron with two spin states: spin antiparallel para-positronium (p-Ps) and spin parallel ortho-positronium (o-Ps).It's known that the intrinsic lifetime of o-Ps via 3γ annihilation under vacuum condition is 142 ns. However, being localized in free volume holes in polymers, o-Ps undergoes 2γ pickup annihilation and its lifetime is shortened down to a few nanoseconds depending on the free volume hole size. Generally, positrons may annihilate via p-Ps, free positron and o-Ps in polymers. These three annihilation branches of positrons can be respectively characterized by their lifetimes τ_1 ($\sim$125 ps), τ_2 ($\sim$300 ps), τ_3 (1$\sim$10 ns) and the corresponding relative intensities. Being the longest lived component, the o-Ps lifetime (τ_{o-Ps}) is of particular importance for the studies of polymer free volumes because it's related to the average free volume hole size and it is determined by the overlapping of positronium wave function with that of the electrons on the wall of free volume holes in the polymer [16–23]. For several decades, PALS has been widely used to measure atom-sized free volume holes in polymers. The average radii of hole free volumes in membranes, which are determined by the semiempirical relationship between τ_{o-Ps} and mean free volume hole radius (R) in a spherical approximation

given by the Tao-Eldrup model as [24,25],

$$\tau_{o-Ps} = 0.5[1 - \frac{R}{R_0} + \frac{1}{2\pi} sin(\frac{2\pi R}{R_0})]^{-1} (ns) \tag{2}$$

where R_0=R+ΔR, and ΔR=0.166 nm is the thickness of the homogeneous electron layer overlapping with the o-Ps wave function. The mean hole free volume V_{FV} can be calculated from the following equation,

$$V_{FV} = \frac{4}{3}\pi R^3. \tag{3}$$

References

[1] NM Ji, OM Jun, CJ Ho, JC Yu, K Woo-Jae, P Juhyun, YW Chang, PJ Yoo, *Soft Matter* **2018**, 10, 1039.

[2] S. Qin, Y. Song, M. E. Floto and J. C. Grunlan, *ACS Appl. Mater. Interfaces* **2017**, 9, 7903-7907.

[3] K. M. Holder, B. R. Spears, M. E. Huff, M. A. Priolo, E. Harth and J. C. Grunlan, *Macromol. Rapid Commun.* **2014**, 35, 960-964.

[4] F. Xiang, S. M. Ward, T. M. Givens and J. C. Grunlan, *ACS Macro Lett.* **2014**, 3, 1055-1058.

[5] C. Cho, F. Xiang, K. L. Wallace and J. C. Grunlan, *Macromolecules* **2015**, 48, 5723-5729.

[6] F. Xiang, T. M. Givens, S. M. Ward and J. C. Grunlan, *ACS Appl. Mater. Interfaces* **2015**, 7, 16148-16151.

[7] Y. Zhan, Y. Meng, Y. Li, C. Zhang, Z. Chen, Poly(vinyl alcohol)/reduced graphene oxide multilayered coatings: the effect of filler content on gas barrier and surface resistivity properties. *Compos. Commun.* **2021**, 24, 100670.

[8] C. Yin, B. Xiong, Q. Liu, J. Li, C. He, *J. Memb. Sci.* **2019**, 591, 117356.

[9] C. Yin, C. He, Q. Liu, B. Xiong, Y. Zhou, *J. Memb. Sci.* **2021**, 625, 119146.

[10] Y.H. Yang, L. Boiling, M.A. Priolo, J.C. Grunlan, *Adv. Mater.* **2013**, 25, 503-508.

[11] H. Liu, T. Kuila, N.H. Kim, B.C. Ku, J.H. Lee, *J. Mater. Chem. A* **2013**, 1, 3739.

[12] J. Fan, W. Zhou, Q. Wang, Z. Chu, L. Yang, L. Yang, J. Sun, L. Zhao, J. Xu, Y. Liang, Z. Chen, *J. Memb. Sci.* **2018**, 549, 581-587.

[13] P. Kirkegaard, N.J. Pedersen, M.M. Eldrup, *J. Electr. Electron. Eng.* **1989**, 5, 152-157.

[14] J. Kansy, *Nucl. Instr. Meth. A* **1996**, 374, 235-244.

[15] C. Yin, J. Li, Y. Zhou, H. Zhang, P. Fang, C. He, *ACS Appl. Mater. Inter.* **2018**, 10, 14026-14035.

[16] Y. Kobayashi, W. Zheng, E. F. Meyer, J. D. McGervey, A. M. Jamieson, R. Simha, *Macromolecules* **1989**, 22, 2302-2306.

[17] J. Liu, Q. Deng, Y. C. Jean, *Macromolecules* 1993, **26**, 7149-7155.

[18] P. N. Patil, D. Roilo, R. S. Brusa, A. Miotello, S. Aghion, R. Ferragut, R. Checchetto, *Phys. Chem. Chem. Phys.* 2016, 18, 3817-3824.

[19] S. K. Sharma, J. Prakash and P. K. Pujari, *Phys. Chem. Chem. Phys.* **2015**, 17, 29201-29209.

[20] C. L. Soles, J. F. Douglas, W. Wu, H. Peng, D. W. Gidley, *Macromolecules* **2004**, 37, 2890-2900.

[21] H. F. M. Mohamed, E. E. Abdel, S. S. Mohamed, *Radiat. Phys. Chem.* **2007**, 76, 160-164.

[22] W. Ma, A. Andersson, J. He, F. H. J. Maure, *Macromolecules* **2008**, 41, 5307-5312.

[23] R. Xia, X. Z. Cao, M. Gao, P. Zhang, M. F. Zeng, B. Y. Wang, L. Wei, *Phys. Chem. Chem. Phys.* **2017**, 19, 3616-3626.

[24] M. Eldrup, D. Lightbody, J. N. Sherwood, *Chem. Phys.* **1981**, 63, 51-58.

[25] S. J. Tao, *J. Chem. Phys.* **1972**, 56, 5499-5510.
